# *N*′-[(*E*)-5-Bromo-2-hy­droxy-3-meth­oxy­benzyl­idene]-4-meth­oxy­benzohydrazide monohydrate

**DOI:** 10.1107/S1600536812033806

**Published:** 2012-09-01

**Authors:** P. R. Reshma, M. Sithambaresan, M. R. Prathapachandra Kurup

**Affiliations:** a Department of Applied Chemistry, Cochin University of Science and Technology, Kochi 682 022, India; b Department of Chemistry, Faculty of Science, Eastern University, Sri Lanka, Chenkalady, Sri Lanka

## Abstract

In the title compound, C_16_H_15_BrN_2_O_4_·H_2_O, the hydrazide mol­ecule is nearly planar, with a largest deviation from the mean plane through the non-H atoms of 0.106 (4) Å and a dihedral angle between the benzene rings of 1.98 (16)°. This mol­ecule adopts an *E* conformation about the C=N bond and an intra­molecular O—H⋯N hydrogen bond increases the rigidity. In the crystal, some mol­ecules of the title hydrazide are replaced by mol­ecules of its 6-bromo isomer, and the Br atom from this admixture mol­ecule was refined to give a partial occupancy of 0.0523 (13). The hydrazide and water mol­ecules are linked through classical N—H⋯O and O—H⋯O hydrogen bonds, forming layers parallel to (110). C—H⋯π inter­actions are also present.

## Related literature

For the biological activity of hydrazone compounds, see: Metwally *et al.* (2006 ▶); Cukurovali *et al.* (2006 ▶). For the synthesis of related compounds, see: Emmanuel *et al.* (2011 ▶); Mangalam & Kurup (2011 ▶). For standard bond lengths, see: Allen *et al.* (1987 ▶). For related structures, see: Tan (2012 ▶); Hou & Bi (2012 ▶); Shen *et al.* (2012 ▶).
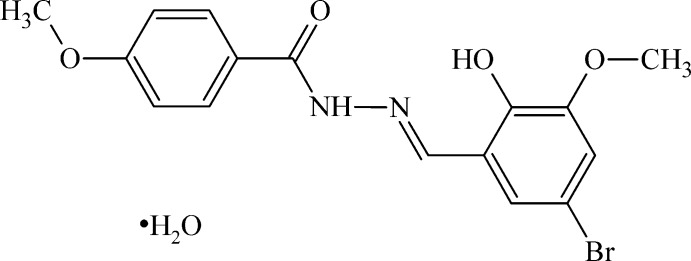


## Experimental

### 

#### Crystal data

C_16_H_15_BrN_2_O_4_·H_2_O
*M* *_r_* = 397.22
Monoclinic, 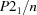

*a* = 4.9730 (4) Å
*b* = 13.5721 (12) Å
*c* = 24.907 (2) Å
β = 90.921 (4)°
*V* = 1680.8 (2) Å^3^
*Z* = 4
Mo *K*α radiation
μ = 2.47 mm^−1^
*T* = 296 K
0.40 × 0.30 × 0.25 mm

#### Data collection

Bruker Kappa APEXII CCD area-detector diffractometer
Absorption correction: multi-scan (*SADABS*; Bruker, 2007 ▶) *T*_min_ = 0.414, *T*_max_ = 0.539
12692 measured reflections
2939 independent reflections
2268 reflections with *I* > 2σ(*I*)
*R*_int_ = 0.036

#### Refinement

*R*[*F* ^2^ > 2σ(*F* ^2^)] = 0.035
*wR*(*F* ^2^) = 0.094
*S* = 1.06
2939 reflections
247 parameters
6 restraints
H atoms treated by a mixture of independent and constrained refinement
Δρ_max_ = 0.52 e Å^−3^
Δρ_min_ = −0.34 e Å^−3^

### 

Data collection: *APEX2* (Bruker, 2007 ▶); cell refinement: *SAINT* (Bruker, 2007 ▶); data reduction: *SAINT*; program(s) used to solve structure: *SHELXS97* (Sheldrick, 2008 ▶); program(s) used to refine structure: *SHELXL97* (Sheldrick, 2008 ▶); molecular graphics: *ORTEP-3* (Farrugia, 1997 ▶) and *DIAMOND* (Brandenburg, 2010 ▶); software used to prepare material for publication: *SHELXL97* and *publCIF* (Westrip, 2010 ▶).

## Supplementary Material

Crystal structure: contains datablock(s) I, global. DOI: 10.1107/S1600536812033806/yk2067sup1.cif

Structure factors: contains datablock(s) I. DOI: 10.1107/S1600536812033806/yk2067Isup2.hkl

Supplementary material file. DOI: 10.1107/S1600536812033806/yk2067Isup3.cml

Additional supplementary materials:  crystallographic information; 3D view; checkCIF report

## Figures and Tables

**Table 1:** Hydrogen-bond geometry (Å, °) *Cg*2 is the centroid of the C9–C14 benzene ring

*D*—H⋯*A*	*D*—H	H⋯*A*	*D*⋯*A*	*D*—H⋯*A*
N2—H21⋯O1*W* ^i^	0.86 (1)	1.96 (2)	2.820 (4)	174 (3)
O2—H2⋯N1	0.87 (3)	1.83 (3)	2.595 (3)	146 (5)
O1*W*—H1*W*⋯O2	0.85 (3)	2.23 (3)	2.974 (4)	147 (4)
O1*W*—H2*W*⋯O3^ii^	0.86 (3)	1.82 (3)	2.675 (3)	177 (4)
C16—H16*C*⋯*Cg*2^iii^	0.96	2.70	3.517 (3)	144

Symmetry codes: (i) 
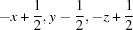
; (ii) 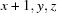; (iii) 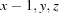.

**Fig. 1.**
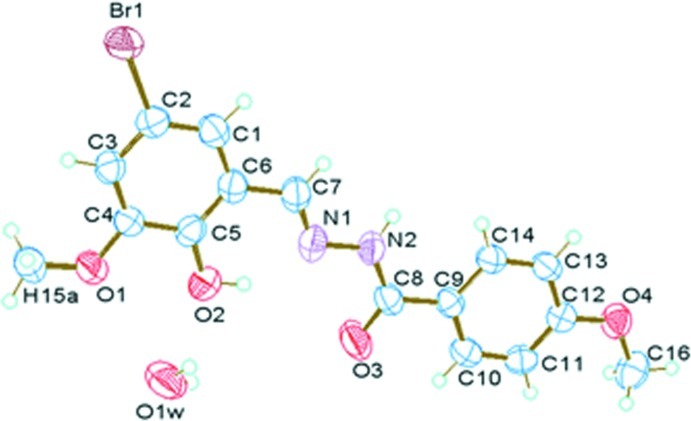
The structure of asymmetric unit of C16H15BrN2O4.H2O with atom labelling scheme and thermal ellipsoids drawn at the 50% probability level. Bromine and hydrogen atoms of the admixture molecule are omitted.

**Fig. 2.**
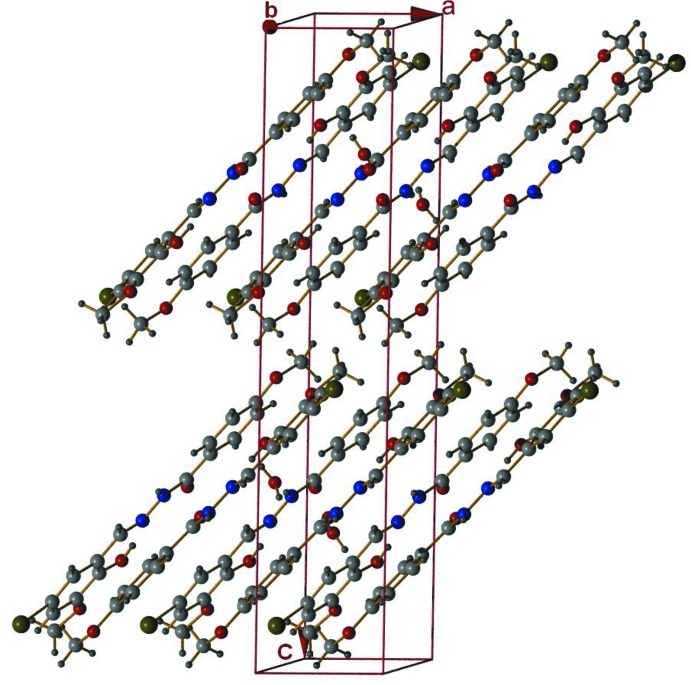
Packing diagram of the title compound.

**Fig. 3.**
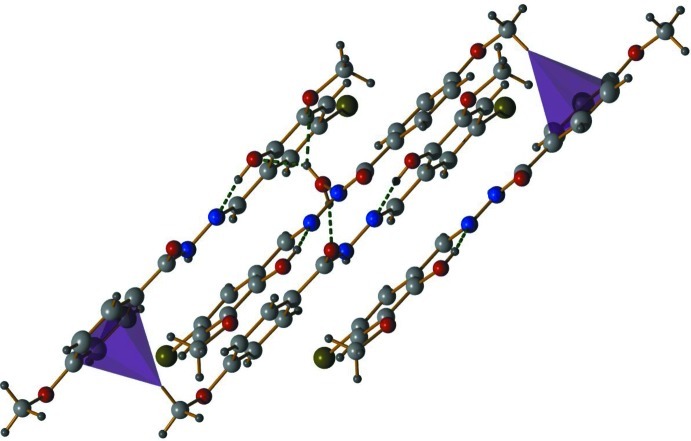
Hydrogen bonds and C—H···π interactions in the crystal structure of C16H15BrN2O4.H2O.

## supplementary crystallographic information

### Comment

Aroylhydrazones derived from the condensation reactions of aroylhydrazides with aldehydes show excellent biological properties (Cukurovali *et al.*, 2006; Metwally *et al.*, 2006). As an extention of the work on the structures of hydrazone derivatives (Tan, 2012; Hou & Bi, 2012; Shen *et al.*, 2012), we report here the crystal structure of a new aroylhydrazone compound. The molecular structure of the title compound is shown in Fig. 1.

The molecule adopts an *E* conformation about the C7═N1 bond and exists in keto form with C8═O3 bond length of 1.216 (2) Å, which is very close to a normal C═O bond length 1.21 Å (Allen *et al.*, 1987).

In the crystal, approximately 5% of molecules of the title hydrazide are replaced by molecules of its 6-bromo isomer, and the Br1B atom of this admixture molecule was included in the refinement. Since the molecule of 6-bromo isomer is likely nonplanar due to sterical tensions, it does not occupy exactly the same position as the molecule of 5-bromo isomer. As a result, Br1B deviates by 0.58 (4) Å from the mean plane of C1–C6 benzene ring, and the distance C1—Br1B is 1.67 (5) Å, that is much smaller than the typical bond length C—Br. On this reason, geometric parameters involving Br1B are not included in the cif-file.

Parallel arrangement of molecules in crystal is shown in Fig. 2. Adjacent molecules are linked through classical N—H···O and O—H···O hydrogen bonds, and a C—H···π interaction between one of the methyl H atoms and the phenyl ring of the adjacent molecule is also observed (see Table 1, Fig. 3). Weak π···π interactions are also present with a shortest separation between benzene ring centroids of 4.973 (3) Å.

### Experimental

The title compound was prepared by adapting a reported procedure (Emmanuel *et al.*, 2011; Mangalam & Kurup, 2011) by refluxing a mixture of methanolic solutions of 4-methoxybenzhydrazide (0.1661 g, 1 mmol) and 5-bromo-3-methoxysalicylaldehyde (0.2309 g, 1 mmol) for 4 h. The formed crystals were collected, washed with few drops of methanol and dried over P_4_O_10_*in vacuo*. Single crystals of the title compound suitable for X-ray analysis were obtained by slow evaporation from its methanolic solution. They contains approximately 5% of the 6-bromo isomer of the title compound.

### Refinement

Bromine atoms Br1A and Br1B were refined freely, with the sum of their occupancy factors constrained to 1.0. All H atoms on C except of H1A and H1B were placed in calculated positions, with C—H bond distances 0.93–0.97 Å and *U*_iso_=1.2Ueq (1.5 for CH_3_). The H1A atom was refined with restrained distance C1—H1A using *DFIX* instruction and with occupancy factor equal to that of Br1A. The H1B was placed in calculated position with occupancy factor equal to that of Br1B, and its coordinates were fixed. Hydrogen atoms attached to O and N atoms were located from difference maps, and the (N,*O*)—H distances were restrained using *DFIX* instructions.

### Crystal data

**Table:**

C_16_H_15_BrN_2_O_4_·H_2_O	*F*(000) = 808
*M**_r_* = 397.22	*D*_x_ = 1.570 Mg m^−^^3^
Monoclinic, *P*2_1_/*n*	Mo *K*α radiation, λ = 0.71073 Å
Hall symbol: -P 2yn	Cell parameters from 4929 reflections
*a* = 4.9730 (4) Å	θ = 2.9–25.6°
*b* = 13.5721 (12) Å	µ = 2.47 mm^−^^1^
*c* = 24.907 (2) Å	*T* = 296 K
β = 90.921 (4)°	Block, brown
*V* = 1680.8 (2) Å^3^	0.40 × 0.30 × 0.25 mm
*Z* = 4	

### Data collection

**Table:**

Bruker Kappa APEXII CCD area-detector diffractometer	2939 independent reflections
Radiation source: fine-focus sealed tube	2268 reflections with *I* > 2σ(*I*)
Graphite monochromator	*R*_int_ = 0.036
Detector resolution: 8.33 pixels mm^-1^	θ_max_ = 25.0°, θ_min_ = 2.9°
ω and φ scan	*h* = −5→5
Absorption correction: multi-scan (*SADABS*; Bruker, 2007)	*k* = −16→16
*T*_min_ = 0.414, *T*_max_ = 0.539	*l* = −29→29
12692 measured reflections	

### Refinement

**Table:**

Refinement on *F*^2^	Primary atom site location: structure-invariant direct methods
Least-squares matrix: full	Secondary atom site location: difference Fourier map
*R*[*F*^2^ > 2σ(*F*^2^)] = 0.035	Hydrogen site location: inferred from neighbouring sites
*wR*(*F*^2^) = 0.094	H atoms treated by a mixture of independent and constrained refinement
*S* = 1.06	*w* = 1/[σ^2^(*F*_o_^2^) + (0.043*P*)^2^ + 0.7865*P*] where *P* = (*F*_o_^2^ + 2*F*_c_^2^)/3
2939 reflections	(Δ/σ)_max_ < 0.001
247 parameters	Δρ_max_ = 0.52 e Å^−^^3^
6 restraints	Δρ_min_ = −0.34 e Å^−^^3^

### Special details

**Table:**

Geometry. All s.u.'s (except the s.u. in the dihedral angle between two l.s. planes) are estimated using the full covariance matrix. The cell s.u.'s are taken into account individually in the estimation of s.u.'s in distances, angles and torsion angles; correlations between s.u.'s in cell parameters are only used when they are defined by crystal symmetry. An approximate (isotropic) treatment of cell s.u.'s is used for estimating s.u.'s involving l.s. planes.
Refinement. Refinement of *F*^2^ against ALL reflections. The weighted *R*-factor *wR* and goodness of fit *S* are based on *F*^2^, conventional *R*-factors *R* are based on *F*, with *F* set to zero for negative *F*^2^. The threshold expression of *F*^2^ > σ(*F*^2^) is used only for calculating *R*-factors(gt) *etc*. and is not relevant to the choice of reflections for refinement. *R*-factors based on *F*^2^ are statistically about twice as large as those based on *F*, and *R*- factors based on ALL data will be even larger.

### Fractional atomic coordinates and isotropic or equivalent isotropic displacement parameters (Å^2^)

**Table:**

	*x*	*y*	*z*	*U*_iso_*/*U*_eq_	Occ. (<1)
O1W	0.6146 (7)	0.53984 (18)	0.20575 (11)	0.0857 (8)	
H1W	0.479 (6)	0.521 (3)	0.1873 (16)	0.129*	
H2W	0.651 (9)	0.496 (2)	0.2293 (14)	0.129*	
Br1A	0.86593 (7)	0.06757 (2)	0.072139 (15)	0.06810 (16)	0.9477 (13)
H1A	0.476 (8)	0.0862 (12)	0.1601 (13)	0.058 (11)*	0.9477 (13)
Br1B	0.5677 (14)	0.0463 (4)	0.1799 (3)	0.064 (3)	0.0523 (13)
H1B	0.7630	0.1200	0.0880	0.058 (11)*	0.0523 (13)
O1	0.5501 (5)	0.43453 (15)	0.08852 (9)	0.0653 (6)	
O2	0.2111 (4)	0.39584 (17)	0.16439 (9)	0.0629 (6)	
O3	−0.2919 (5)	0.39947 (18)	0.27910 (10)	0.0789 (7)	
O4	−1.0445 (4)	0.21328 (16)	0.45317 (8)	0.0616 (6)	
N1	0.0049 (5)	0.2680 (2)	0.22932 (9)	0.0554 (6)	
N2	−0.1688 (5)	0.2415 (2)	0.26906 (9)	0.0528 (6)	
C1	0.4937 (6)	0.1490 (2)	0.14527 (12)	0.0534 (7)	
C2	0.6652 (6)	0.1699 (2)	0.10475 (11)	0.0502 (7)	
C3	0.6936 (6)	0.2646 (2)	0.08475 (11)	0.0514 (7)	
H3	0.8149	0.2775	0.0576	0.062*	
C4	0.5407 (6)	0.3389 (2)	0.10554 (11)	0.0489 (7)	
C5	0.3586 (5)	0.3192 (2)	0.14656 (11)	0.0478 (7)	
C6	0.3366 (6)	0.2237 (2)	0.16659 (11)	0.0492 (7)	
C7	0.1512 (6)	0.1998 (2)	0.20906 (11)	0.0559 (7)	
H7	0.1382	0.1354	0.2216	0.067*	
C8	−0.3172 (6)	0.3134 (2)	0.29184 (11)	0.0511 (7)	
C9	−0.5107 (5)	0.2833 (2)	0.33332 (10)	0.0444 (6)	
C10	−0.6695 (6)	0.3560 (2)	0.35495 (12)	0.0514 (7)	
H10	−0.6532	0.4202	0.3424	0.062*	
C11	−0.8522 (6)	0.3363 (2)	0.39472 (12)	0.0514 (7)	
H11	−0.9568	0.3866	0.4088	0.062*	
C12	−0.8775 (5)	0.2411 (2)	0.41334 (11)	0.0459 (7)	
C13	−0.7265 (6)	0.1670 (2)	0.39097 (12)	0.0530 (7)	
H13	−0.7489	0.1024	0.4025	0.064*	
C14	−0.5433 (6)	0.1873 (2)	0.35177 (12)	0.0515 (7)	
H14	−0.4406	0.1367	0.3375	0.062*	
C15	0.7560 (7)	0.4612 (2)	0.05349 (14)	0.0685 (9)	
H15A	0.9270	0.4437	0.0692	0.103*	
H15B	0.7503	0.5310	0.0473	0.103*	
H15C	0.7317	0.4272	0.0200	0.103*	
C16	−1.2105 (6)	0.2868 (3)	0.47648 (13)	0.0702 (9)	
H16A	−1.0998	0.3387	0.4908	0.105*	
H16B	−1.3132	0.2581	0.5048	0.105*	
H16C	−1.3305	0.3131	0.4495	0.105*	
H21	−0.165 (7)	0.1796 (9)	0.2766 (13)	0.067 (10)*	
H2	0.116 (8)	0.375 (4)	0.1910 (13)	0.130 (18)*	

### Atomic displacement parameters (Å^2^)

**Table:**

	*U*^11^	*U*^22^	*U*^33^	*U*^12^	*U*^13^	*U*^23^
O1W	0.122 (2)	0.0518 (14)	0.0830 (19)	−0.0127 (14)	−0.0071 (16)	0.0124 (12)
Br1A	0.0682 (2)	0.0490 (2)	0.0875 (3)	0.00715 (17)	0.01531 (18)	−0.00284 (18)
Br1B	0.080 (5)	0.040 (4)	0.072 (5)	−0.002 (3)	−0.010 (3)	0.004 (3)
O1	0.0735 (14)	0.0462 (12)	0.0770 (14)	0.0028 (10)	0.0238 (12)	0.0056 (11)
O2	0.0632 (14)	0.0619 (13)	0.0643 (14)	0.0019 (11)	0.0177 (11)	−0.0062 (11)
O3	0.0930 (18)	0.0588 (14)	0.0854 (17)	0.0022 (13)	0.0209 (14)	0.0284 (12)
O4	0.0562 (12)	0.0676 (14)	0.0615 (12)	0.0041 (11)	0.0159 (10)	0.0053 (11)
N1	0.0516 (14)	0.0730 (18)	0.0415 (13)	−0.0144 (13)	0.0024 (11)	0.0006 (12)
N2	0.0566 (15)	0.0569 (17)	0.0453 (13)	−0.0085 (13)	0.0100 (11)	0.0035 (12)
C1	0.0563 (18)	0.0503 (18)	0.0536 (17)	−0.0071 (15)	0.0001 (14)	0.0039 (15)
C2	0.0459 (16)	0.0512 (17)	0.0536 (17)	−0.0003 (13)	−0.0002 (13)	−0.0052 (14)
C3	0.0462 (16)	0.0546 (18)	0.0535 (17)	−0.0059 (14)	0.0074 (13)	−0.0012 (14)
C4	0.0508 (16)	0.0435 (16)	0.0526 (16)	−0.0040 (13)	0.0049 (13)	−0.0019 (13)
C5	0.0460 (15)	0.0545 (18)	0.0431 (15)	−0.0040 (13)	0.0025 (12)	−0.0071 (13)
C6	0.0466 (16)	0.0577 (18)	0.0434 (15)	−0.0107 (14)	−0.0012 (12)	−0.0007 (13)
C7	0.0575 (18)	0.0621 (19)	0.0481 (16)	−0.0116 (16)	0.0041 (14)	0.0027 (15)
C8	0.0568 (17)	0.0482 (18)	0.0483 (16)	−0.0048 (14)	−0.0034 (13)	0.0089 (14)
C9	0.0444 (15)	0.0444 (15)	0.0442 (15)	−0.0014 (12)	−0.0037 (12)	0.0050 (12)
C10	0.0540 (17)	0.0422 (16)	0.0579 (17)	0.0009 (13)	−0.0047 (14)	0.0044 (13)
C11	0.0476 (16)	0.0480 (17)	0.0586 (18)	0.0083 (13)	−0.0014 (14)	−0.0048 (14)
C12	0.0390 (14)	0.0509 (17)	0.0478 (15)	0.0006 (12)	−0.0022 (12)	0.0001 (13)
C13	0.0577 (17)	0.0416 (16)	0.0600 (18)	−0.0024 (13)	0.0070 (14)	0.0041 (13)
C14	0.0557 (17)	0.0428 (16)	0.0562 (17)	0.0023 (13)	0.0092 (14)	0.0008 (13)
C15	0.074 (2)	0.0533 (19)	0.079 (2)	−0.0056 (16)	0.0200 (18)	0.0094 (17)
C16	0.0533 (19)	0.093 (3)	0.065 (2)	0.0132 (18)	0.0134 (16)	−0.0010 (18)

### Geometric parameters (Å, º)

**Table:**

O1W—H1W	0.85 (3)	C5—C6	1.394 (4)
O1W—H2W	0.86 (3)	C6—C7	1.451 (4)
Br1A—C2	1.901 (3)	C7—H7	0.9300
O1—C4	1.366 (3)	C8—C9	1.480 (4)
O1—C15	1.404 (4)	C9—C10	1.379 (4)
O2—C5	1.352 (3)	C9—C14	1.393 (4)
O2—H2	0.87 (3)	C10—C11	1.381 (4)
O3—C8	1.217 (3)	C10—H10	0.9300
O4—C12	1.357 (3)	C11—C12	1.379 (4)
O4—C16	1.425 (4)	C11—H11	0.9300
N1—C7	1.285 (4)	C12—C13	1.378 (4)
N1—N2	1.372 (3)	C13—C14	1.374 (4)
N2—C8	1.353 (4)	C13—H13	0.9300
N2—H21	0.861 (14)	C14—H14	0.9300
C1—C2	1.362 (4)	C15—H15A	0.9600
C1—C6	1.390 (4)	C15—H15B	0.9600
C1—H1A	0.93 (4)	C15—H15C	0.9600
C2—C3	1.386 (4)	C16—H16A	0.9600
C3—C4	1.370 (4)	C16—H16B	0.9600
C3—H3	0.9300	C16—H16C	0.9600
C4—C5	1.402 (4)		
			
H1W—O1W—H2W	108 (2)	N2—C8—C9	117.3 (2)
C4—O1—C15	117.8 (2)	C10—C9—C14	118.0 (3)
C5—O2—H2	108 (3)	C10—C9—C8	117.3 (2)
C12—O4—C16	117.9 (2)	C14—C9—C8	124.7 (3)
C7—N1—N2	117.5 (3)	C9—C10—C11	122.0 (3)
C8—N2—N1	117.9 (3)	C9—C10—H10	119.0
C8—N2—H21	128 (2)	C11—C10—H10	119.0
N1—N2—H21	114 (2)	C12—C11—C10	119.2 (3)
C2—C1—C6	119.6 (3)	C12—C11—H11	120.4
C2—C1—H1A	123 (2)	C10—C11—H11	120.4
C6—C1—H1A	117 (2)	O4—C12—C13	115.9 (3)
C1—C2—C3	121.9 (3)	O4—C12—C11	124.5 (3)
C1—C2—Br1A	120.3 (2)	C13—C12—C11	119.6 (3)
C3—C2—Br1A	117.8 (2)	C14—C13—C12	120.8 (3)
C4—C3—C2	119.1 (3)	C14—C13—H13	119.6
C4—C3—H3	120.5	C12—C13—H13	119.6
C2—C3—H3	120.5	C13—C14—C9	120.3 (3)
O1—C4—C3	124.1 (2)	C13—C14—H14	119.9
O1—C4—C5	115.7 (2)	C9—C14—H14	119.9
C3—C4—C5	120.3 (3)	O1—C15—H15A	109.5
O2—C5—C6	123.5 (2)	O1—C15—H15B	109.5
O2—C5—C4	116.9 (3)	H15A—C15—H15B	109.5
C6—C5—C4	119.6 (3)	O1—C15—H15C	109.5
C1—C6—C5	119.6 (3)	H15A—C15—H15C	109.5
C1—C6—C7	118.9 (3)	H15B—C15—H15C	109.5
C5—C6—C7	121.5 (3)	O4—C16—H16A	109.5
N1—C7—C6	119.7 (3)	O4—C16—H16B	109.5
N1—C7—H7	120.1	H16A—C16—H16B	109.5
C6—C7—H7	120.1	O4—C16—H16C	109.5
O3—C8—N2	121.5 (3)	H16A—C16—H16C	109.5
O3—C8—C9	121.2 (3)	H16B—C16—H16C	109.5
			
C7—N1—N2—C8	177.9 (3)	C1—C6—C7—N1	179.8 (3)
C6—C1—C2—C3	1.7 (5)	C5—C6—C7—N1	−0.7 (4)
C6—C1—C2—Br1A	−176.3 (2)	N1—N2—C8—O3	−2.6 (4)
C1—C2—C3—C4	−1.4 (4)	N1—N2—C8—C9	177.9 (2)
Br1A—C2—C3—C4	176.5 (2)	O3—C8—C9—C10	3.1 (4)
C15—O1—C4—C3	−10.0 (4)	N2—C8—C9—C10	−177.4 (2)
C15—O1—C4—C5	170.6 (3)	O3—C8—C9—C14	−177.1 (3)
C2—C3—C4—O1	−179.3 (3)	N2—C8—C9—C14	2.4 (4)
C2—C3—C4—C5	0.1 (4)	C14—C9—C10—C11	1.6 (4)
O1—C4—C5—O2	0.6 (4)	C8—C9—C10—C11	−178.5 (3)
C3—C4—C5—O2	−178.8 (3)	C9—C10—C11—C12	−0.2 (4)
O1—C4—C5—C6	−179.7 (3)	C16—O4—C12—C13	−178.2 (3)
C3—C4—C5—C6	0.9 (4)	C16—O4—C12—C11	1.8 (4)
C2—C1—C6—C5	−0.6 (4)	C10—C11—C12—O4	178.2 (3)
C2—C1—C6—C7	178.8 (3)	C10—C11—C12—C13	−1.9 (4)
O2—C5—C6—C1	179.0 (3)	O4—C12—C13—C14	−177.5 (3)
C4—C5—C6—C1	−0.6 (4)	C11—C12—C13—C14	2.5 (4)
O2—C5—C6—C7	−0.4 (4)	C12—C13—C14—C9	−1.1 (4)
C4—C5—C6—C7	179.9 (3)	C10—C9—C14—C13	−0.9 (4)
N2—N1—C7—C6	179.5 (2)	C8—C9—C14—C13	179.2 (3)

### Hydrogen-bond geometry (Å, º)

Cg2 is the centroid of the C9–C14 benzene ring

**Table:**

*D*—H···*A*	*D*—H	H···*A*	*D*···*A*	*D*—H···*A*
N2—H21···O1*W*^i^	0.86 (1)	1.96 (2)	2.820 (4)	174 (3)
O2—H2···N1	0.87 (3)	1.83 (3)	2.595 (3)	146 (5)
O1*W*—H1*W*···O2	0.85 (3)	2.23 (3)	2.974 (4)	147 (4)
O1*W*—H2*W*···O3^ii^	0.86 (3)	1.82 (3)	2.675 (3)	177 (4)
C16—H16*C*···*Cg*2^iii^	0.96	2.70	3.517 (3)	144

Symmetry codes: (i) −*x*+1/2, *y*−1/2, −*z*+1/2; (ii) *x*+1, *y*, *z*; (iii) *x*−1, *y*, *z*.
